# Statistical learning in social action contexts

**DOI:** 10.1371/journal.pone.0177261

**Published:** 2017-05-05

**Authors:** Claire Monroy, Marlene Meyer, Sarah Gerson, Sabine Hunnius

**Affiliations:** 1 Donders Institute for Brain, Cognition and Behaviour, Radboud University Nijmegen, Nijmegen, Netherlands; 2 Department of Otolaryngology–Head and Neck Surgery, Ohio State University Wexner Medical Center, Columbus, Ohio, United States; 3 Department of Psychology, University of Chicago, Chicago, Illinois, United States; 4 School of Psychology, Cardiff University, Cardiff, Wales; Vanderbilt University, UNITED STATES

## Abstract

Sensitivity to the regularities and structure contained within sequential, goal-directed actions is an important building block for generating expectations about the actions we observe. Until now, research on statistical learning for actions has solely focused on individual action sequences, but many actions in daily life involve multiple actors in various interaction contexts. The current study is the first to investigate the role of statistical learning in tracking regularities between actions performed by different actors, and whether the social context characterizing their interaction influences learning. That is, are observers more likely to track regularities across actors if they are perceived as acting jointly as opposed to in parallel? We tested adults and toddlers to explore whether social context guides statistical learning and—if so—whether it does so from early in development. In a between-subjects eye-tracking experiment, participants were primed with a social context cue between two actors who either shared a goal of playing together (‘Joint’ condition) or stated the intention to act alone (‘Parallel’ condition). In subsequent videos, the actors performed sequential actions in which, for certain action pairs, the first actor’s action reliably predicted the second actor’s action. We analyzed predictive eye movements to upcoming actions as a measure of learning, and found that both adults and toddlers learned the statistical regularities across actors when their actions caused an effect. Further, adults with high statistical learning performance were sensitive to social context: those who observed actors with a shared goal were more likely to correctly predict upcoming actions. In contrast, there was no effect of social context in the toddler group, regardless of learning performance. These findings shed light on how adults and toddlers perceive statistical regularities across actors depending on the nature of the observed social situation and the resulting effects.

## Introduction

### Statistical learning of action sequences

Statistical learning refers to the fundamental ability to extract regularities from continuous sensory input. These skills support learning in multiple domains and across development from early in infancy throughout adulthood [1–3]. Observers and listeners can, for instance, readily extract regularities from sequences of visual shapes [4], auditory tones [5], linguistic items, and grammatical structures (for an in-depth discussion, see [6]). The range and scope of this mechanism has led to the view that statistical learning forms part of the basic cognitive skill set necessary for language acquisition [6] and understanding of mental states [7]. Recently, researchers have demonstrated that statistical learning also extends to the action domain. Across development, humans are sensitive to the regularities and structure contained within sequential, goal-directed actions they observe others perform [8–9]. For instance, even 10- to 11-month-old infants notice when familiar action streams, such as someone cleaning a kitchen, are interrupted with pauses that disrupt the known structure of the sequence [10].

Evidence for statistical learning in the action domain largely comes from studies of individual action sequences performed by a single actor. Observers are sensitive to multiple sources of statistical information within structured action sequences, such as the transitional probabilities and co-occurrence frequencies between action steps [8–11]. Adults and infants make use of this structure to segment continuous action streams into discrete events [11] and to make predictions about the goal-directed behavior of solo actors [9].

Yet, many everyday action sequences occur in a social context and are performed by multiple, rather than individual, actors. For instance, two people wash dishes together by coordinating their movements as one person soaps, scrubs, and rinses the dishes and the second person dries and puts them away. Predicting and understanding the overall joint goal of such actions entails tracking the regularities, not only within one person’s actions, but across both actors. In the given example, to anticipate what will happen next, one needs to know the likelihood of what the second actor (the dish dryer) will do given what the first actor is currently doing (rinsing a dish). Experienced observers automatically and implicitly make anticipatory eye movements that reflect their predictions of how familiar individual actions will unfold [12]; for instance, when watching an actor place a ball into a bucket, observers will look at the bucket predictively before the actor’s hand arrives there. For a naïve observer, such as a young infant, learning to predict the upcoming events in the sequence indicates recognition of the actors’ overarching collaborative goal and each of their individual aims. However, it is unknown whether and how we predict the interleaved actions of multiple people.

### Social context: Joint versus parallel action

Joint actions have been broadly defined as “any form of social interaction whereby two or more individuals coordinate their actions in space and time to bring about a change in the environment” (p.70, [13]). Two people’s actions can be coordinated in time and space with or without a common goal that characterizes their respective actions. For instance, two runners on the same path in Central Park might be coordinated in space and time, but their actions have independent, or ‘parallel’, goals. In contrast, two people who wash dishes together share the joint goal to clean. Researchers have classified collaborative actions on the basis of the underlying goals of the two actors [14, 15]. In the current study, we adopt the distinction between ‘joint’ and ‘parallel’ actions based on whether or not they share a common goal (for a detailed discussion of this topic, see [15]).

An observer’s perception of goal-directed interactions between two people varies depending on whether he or she thinks that the actors are pursuing joint or parallel goals. In a recent study, Eskenazi and colleagues [16] compared neural responses during observation of two perceptually identical scenes in which two actors engaged together in a task. The scenes were preceded by a social cue that established whether the actors in the video had joint or parallel goals. Findings revealed that the observation of these actions activated different neural regions depending on the goal of the actors, even though the actions were perceptually identical. Converging evidence from a separate line of research shows that communicative social interactions between two people helps observers anticipate visual elements of the unfolding activity and ascribe particular goals to the individual actors [17].

The importance of social context for learning early in development is well-established [18–20]. In a recent study, Fawcett and Gredebäck [21] showed toddlers movies in which an actor moved a block to an intermediary location and a second actor then moved the block to a final location. When the first actor subsequently appeared alone, toddlers spontaneously anticipated that she would move the block to the final goal location, thus assuming that she shared the goal of moving the block to this location with the second actor. Crucially, they did so only if the actors had previously engaged in a social interaction, indicating that social context guided the toddlers’ predictions about the action outcome. This sensitivity appears to emerge after 18 months of age [21] as younger infants could not do so in a follow-up study [22]. Other research has shown that 9-month-olds infants can, however, use simple social cues such as eye gaze to help them extract regularities amidst distracting patterns [23]. Learning statistical regularities is considered a fundamental building block for social understanding [7], yet there is limited evidence for a direct role of statistical learning in a social action context during development or in adulthood. One possibility, based on these previous studies, is that the social goals underlying interactions between people shape the statistical information that observers extract and retain during action observation.

### The effects of goal-directed actions

The sensory consequences of actions, typically called *action-effects*, co-occur with goal-directed actions and are another important feature of the action context. Actions naturally cause effects that are associated with an action goal or outcome, such as the appearance of a light when switching on a lamp. Adults and infants readily acquire bidirectional associations between these effects and the motor plans that produce them [24, 25]. Action-effect associations support learning about action goals for both observed and self-produced actions, making them particularly important as young children learn novel goal-directed actions and the functions of corresponding objects [26–28].

However, research on acquisition of action-effect couplings within continuous action sequences is limited. A recent study showed that action-effects were necessary for toddlers to transfer learned actions (of a single actor) into their own action choices, possibly because the effects were perceived as a desirable outcome or a goal [9]. Action-effects could also guide attention to the regularities in sequential actions across multiple actors, although to our knowledge the current study is the first to test this empirically. The ability to learn action-effect couplings, when caused by two co-actors, may be easier when those actors are observed to share a joint goal.

### The current experiment

We conducted an eye-tracking experiment with adults and 18-month-old toddlers to assess whether social context moderates statistical learning during observation of action sequences. Our primary aim was to establish whether observers can track statistical regularities that occur across actions of multiple actors. That is, can they predict the actions of one actor based on the previous action of another actor? Secondly, we asked whether statistical regularities distributed across actors are more readily learned when these actors share a joint goal. We hypothesized that observers would be better at learning—as measured by anticipatory gaze fixations and action performance—when actions between two individuals are perceived as a joint sequence with a shared goal between the actors. Further, we expected that action-effects would generally facilitate learning but that observers would benefit most from this cue in a joint action context. Based on prior evidence from Eskenazi et al. [16] and Fawcett and Gredebäck [21], we hypothesized that both adults and 18-month-old toddlers would demonstrate a similar pattern. We measured predictive gaze fixations during an action observation phase, followed by an action performance phase in which participants performed their own action sequences both alone and together with an experimenter.

## Method

### Participants

Sixty adults and 58 toddlers took part in this study. Toddlers were recruited from a database of interested families in the surrounding region. Ten of the adults and 14 of the toddlers were excluded from final analyses for failing to meet a minimum looking time requirement (see *Analysis* section) resulting in 50 adults and 44 toddlers in the final sample (see Table 1 for mean ages and condition assignments). Written informed consent was obtained from all adult participants and parents of the toddlers. Adults received course credit for their participation, and families received a baby book or 10€ as a gift. This study was approved by the local ethics committee (Ethische Commissie Gedragswetenschappelijk Onderzoek; ECG2012-1301-006 Stapel/Hunnius).

**Table 1:** Characteristics of the final sample.

Age Group	*N*	*Mean Age (SD)*
Adults	50	22.59 (*3*.*82*) years
Joint	23	22.18 (*3*.*11*)
Parallel	27	22.94 (*4*.*36*)
Toddlers	44	17.00 (.*26*) months
Joint	24	17.94 (.*26*)
Parallel	20	18.04 (.*24*)

### Stimuli

**Fig 1:**
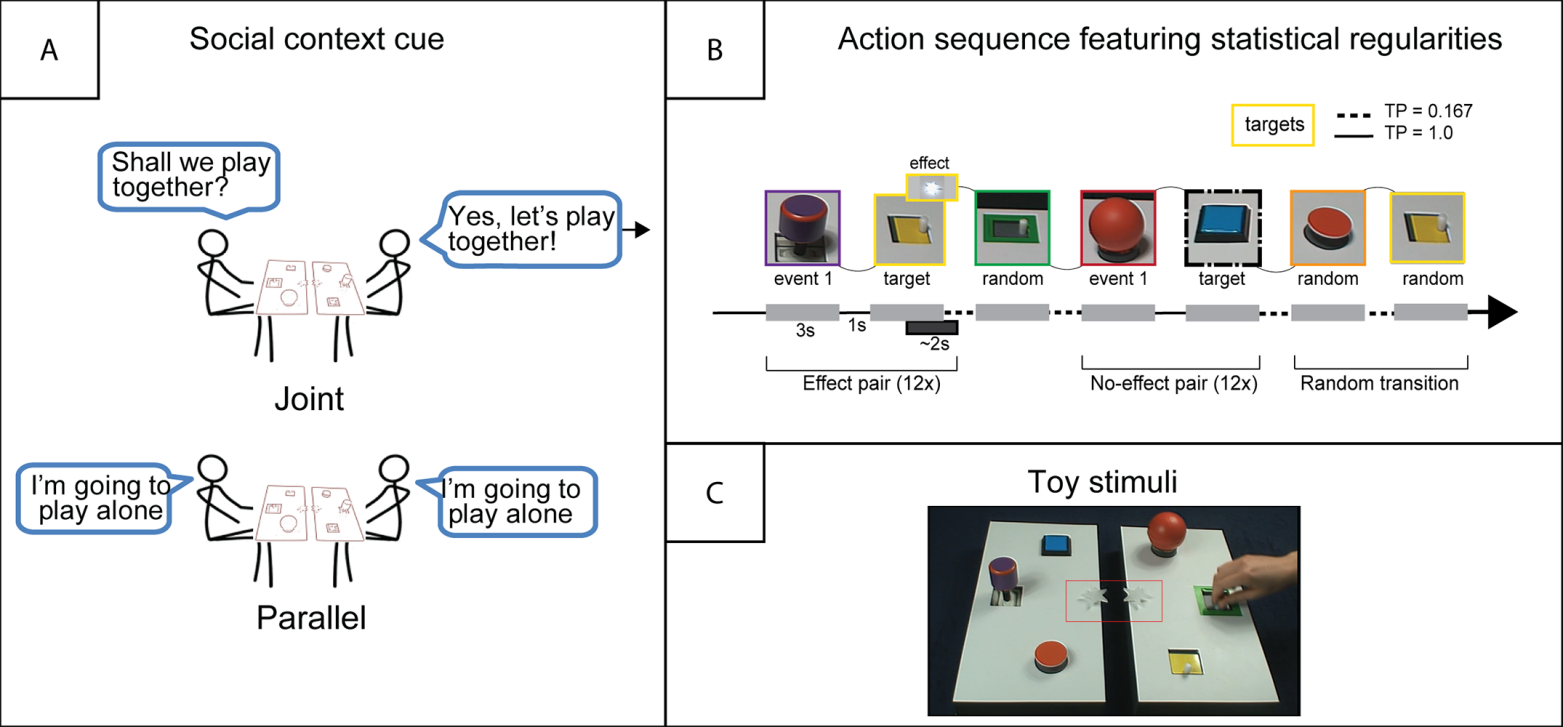
Schematic illustrating the experimental design. (A) Participants first observed either a Joint or a Parallel social cue film.(B) Both conditions subsequently observed an identical action sequence featuring deterministic action pairs embedded within a random sequence. Targets (outlined in yellow) are the second actions of deterministic pairs which occurred within pairs (Deterministic trials) or randomly (Random trials). (C) Still frame from an action sequence video depicting the toybox stimulus. The location of the light effects is outlined in red.

We created two complementary rectangular toys that were arranged approximately 3cm apart from one another. Each rectangle contained three unique objects with distinct manual affordances, and a star-shaped light as the effect (Fig 1). Lights were controlled by two external buttons and could be activated at any time by the experimenter. Video stimuli of actions performed with the toys were filmed with a Sony HandyCam video camera and edited using Adobe Premiere Pro Cs5 software.

### Social context cue

Participants were randomly assigned to either a Joint or a Parallel condition which determined the social context of the observed actions. They first observed a brief social cue film depicting two actors seated at a table with the toy placed on the table between them. In both conditions, the actors first faced each other and engaged in a brief conversation about interacting with the toy (Fig 1A). Similar to the social cues used in previous research [21], the conditions differed in the orientation and eye-contact between the actors and the statements they made. In the Joint condition, the actors first made direct eye contact with each other before saying “Shall we play together?” (Actor 1) and “Yes, let’s play together!” (Actor 2). In the Parallel condition, they instead faced slightly away from each other and each stated “I am going to play alone” (Actor 1, then Actor 2). At the end of each film, the camera zoomed into the toy as though the social clip and the action sequence were one continuous demonstration. The social cue movie clips were 10s long and repeated before each experimental block throughout the action sequence (described below).

### Action sequence

After the social cue, participants watched videos of the actors performing action sequences with the toy stimuli, in which only their hands were visible (see Fig 1C). Individual actions were defined as the manipulation of one object by an actor, who always acted upon the rectangular half of the toy closest to her.

Two pseudo-randomized action sequences were generated using the program Mix (van Casteren, 2006). Sequences were defined according to the transitional probabilities between individual actions directed at the objects on the toy (e.g. ‘push’, ‘pull’, etc.). Two action pairs featured deterministic transitions: action ‘A’ followed action ‘B’ with 100% probability (e.g., ‘push’ always followed ‘pull’). Transitions between all other unpaired actions featured a 0.167 (1/6) transitional probability (Fig 1B). Crucially, the two actions that formed an action pair were each performed on opposite rectangular parts of the toy. For instance, if the first action was performed by Actor 1 on the toy closest to her, the second action would always be performed by Actor 2 on the opposite toy. Thus, the statistical regularities that could be learned always occurred across the two actors.

One of the deterministic action pairs (Effect pair) caused a light to turn on during the second action; the second pair (No-effect pair) did not cause any effect. The activated light was always on the same half of the toy as its corresponding action and thus opposite to the first action of the Effect pair (Fig 1C). For both the Effect and No-effect pair, we defined the second actions as *target* actions, as these were the actions that became predictable as the sequence unfolded. All actions occurred with equal frequency with the exception of target actions: targets occurred within pairs (12 times) and also at random (12 times) following any other possible action (random trials). During random trials, target actions associated with the Effect pair never activated the light. This ensured that participants needed to learn the two-step action pair in order to predict the effect, as it did not consistently follow the target action. The actions that defined the Effect and No-effect pairs were randomly selected and counterbalanced across participants. Lastly, no action or pair could occur more than three times consecutively.

Video sequences were divided into four blocks. The orientation of the toys (Fig 1C) rotated 180° between blocks to ensure that individuals had to base their predictive looks on an action rather than a location. Action sequences were interleaved with social cue clips which were repeated at the beginning of each block. In total, the entire action observation phase lasted approximately eight minutes. Engaging, upbeat music was played to help sustain toddlers’ attention throughout the sequences and was played for the adults to maintain consistency. The music did not correspond in any way to the unfolding actions.

### Procedure

The experiment consisted of an action observation phase using an eye-tracker, followed by an action performance phase in which participants’ own actions were videotaped and coded offline (using the same toys from the video stimuli). After obtaining written informed consent, participants were seated on a chair approximately 60cm from the eye-tracker screen. Toddlers were seated on their parent’s lap during all phases of the experiment.

A 9-point calibration sequence was repeated until valid data was acquired for all nine calibration points or for a maximum of three attempts. Following calibration, participants were shown the video demonstration as described above. Parents were instructed to look away during calibration and to refrain from influencing their child during the experiment. Participants’ eye movements were recorded with a Tobii T120 eye-tracker (Tobii, Stockholm, Sweden) with a 17” monitor (1280x1024 pixels) and sampling frequency of 60Hz. Stimuli were presented with Tobii Studio 2.0 presentation software and sounds were played through external speakers.

After the videos were completed, participants moved to a nearby table for the action performance phase and were presented with the two rectangular toys. The action performance phase consisted of two parts: an Individual and a Joint phase. In the Individual phase, participants were told that they could now play with the toys however they liked and were given 90 seconds to perform their own action sequences.

In the Joint phase, the experimenter and participant took turns acting with the toys for a total of 12 turns each: the experimenter would perform an action and then the participant was encouraged to perform one action. The experimenter performed actions on the toy piece closest to her in a pseudorandom but pre-selected order, in which she always performed the first action of the Effect and No-effect pairs three times each (i.e., a 25% probability for each pair). She never performed the target actions. After the first six trials, the toy pieces were rotated and the final six trials were repeated in the same way.

The order of action performance phases (Individual and Joint) was counterbalanced across participants. A camera recorded this session and behavior was later coded offline. Throughout both phases, the experimenter sat opposite the participant and pressed a hidden button to activate the light if the participant performed the Effect pair.

### Data analysis

#### Eye-tracking data

Adult and toddler eye-tracking data were processed in identical ways and were analyzed separately. To ensure sufficient looking time to learn the statistical structure, we did not include those individuals with total fixation time of more than one standard deviation below the mean within each age group. Toddlers below this criterion watched the videos for a mean of 34.52 seconds, which corresponds to less than 10% of the demonstration and less than one observation of each pair. For consistency, we applied the same criterion to the adult group as well; this excluded adults who contributed gaze data for less than 15% of the demonstration. This resulted in the exclusion of 10 adults and 12 toddlers, as noted above in the *Participants* section.

Gaze fixations were extracted from the raw eye-tracking data using a custom-made program with a temporal filter of 100ms and a spatial filter of 30 pixels. Fixation data were imported into Matlab for further analysis. Fixations were considered anticipatory if they occurred during predictive time windows, which began the moment a hand appeared to perform the first action of a deterministic pair until just before it reappeared to perform the target action. This represents the time window during which the observer could predict the next action before it actually occurred. The first action pair was excluded from analyses, as participants could not make a prediction based on prior information during the first observation.

Equally-sized regions of interest (ROIs) were defined around each object (250 square pixels). If participants learned the sequential pair structure, they should look more to the target object of each pair during predictive time windows than to any other object. Fixations in the ROI of the target action were considered correct. Fixations in the ROI of the ongoing action (the first action of each pair) were excluded. Fixations to the star were counted as correct during the Effect pair and were excluded entirely for the No-effect pair. For incorrect fixations, we summed the total fixations to the four alternative locations and divided by four to yield the average number of fixations to an incorrect region. Our dependent measures were the proportions of correct and incorrect fixations, out of the total predictive fixations, averaged across the predictive windows for all target trials (Eqs 1–4).

Effect Pair:

$$Correct=\;\frac{\#\;looks\;to\;target\;\&\;effect}{total\;\#\;looks\;to\;all\;objects\;\&\;effect}$$ (1)

$$Incorrect=\;\frac{\#\;looks\;to\;other\;4\;objects/4}{total\;\#\;looks\;to\;all\;objects\;\&\;effect}$$ (2)

No-effect Pair:

$$Correct=\;\frac{\#\;looks\;to\;target}{total\;\#\;looks\;to\;all\;objects}$$ (3)

$$Incorrect=\;\frac{\#\;looks\;to\;other\;4\;objects/4}{total\;\#\;looks\;to\;all\;objects}$$ (4)

### Learning performance

In the statistical learning literature, not all individuals demonstrate learning—across various performance measures—and there is typically variability in the degree to which regularities are learned [e.g. [2, 29–31]. A primary aim of the current study was to investigate whether social information directly guides statistical learning in a top-down manner [17, 32]. However, participants must demonstrate the ability to learn the regularities in the action sequences at all, before social cues can be expected to exert any influence on how well they are able to encode them. To investigate relations between the degree of learning and social contexts, we categorized adults and toddlers into two sub-groups based on their learning performance. Learning performance was assessed by computing a difference score for each participant of *Correct–Incorrect* gaze proportions for each pair. We hypothesized that learning performance would be related to the social context that they observed. Specifically, we expected that among the participants with higher difference scores, those who observed the Joint condition should demonstrate higher performance than those who observed the Parallel condition.

### Action performance data

For the Individual and Joint phases, video recordings were coded offline to assess the sequence of actions that each participant performed after initiating contact with the toy. If participants performed two actions simultaneously (i.e. using both hands), both actions were coded. Any manipulation of an object was considered an action. As our hypotheses were not related to the kinematics of the actions, but rather their sequential order, we ignored individual differences in kinematics and only assessed the order of actions performed with the respective objects. Next, we calculated the conditional probabilities of performing each action pair for each participant. For clarity, we will refer to the first action of a pair as ‘*A*’ and the second, target action of a pair as ‘*B*’. The conditional probability of performing *B*, given the performance of *A* by either the participant (Individual phase) or experimenter (Joint phase), is defined as:

$$P\left(B|A\right)=\;\frac{P\left(A\;and\;B\right)}{P\left(A\right)}$$

## Results

### Adults

#### Eye-tracking results

**Fig 2:**
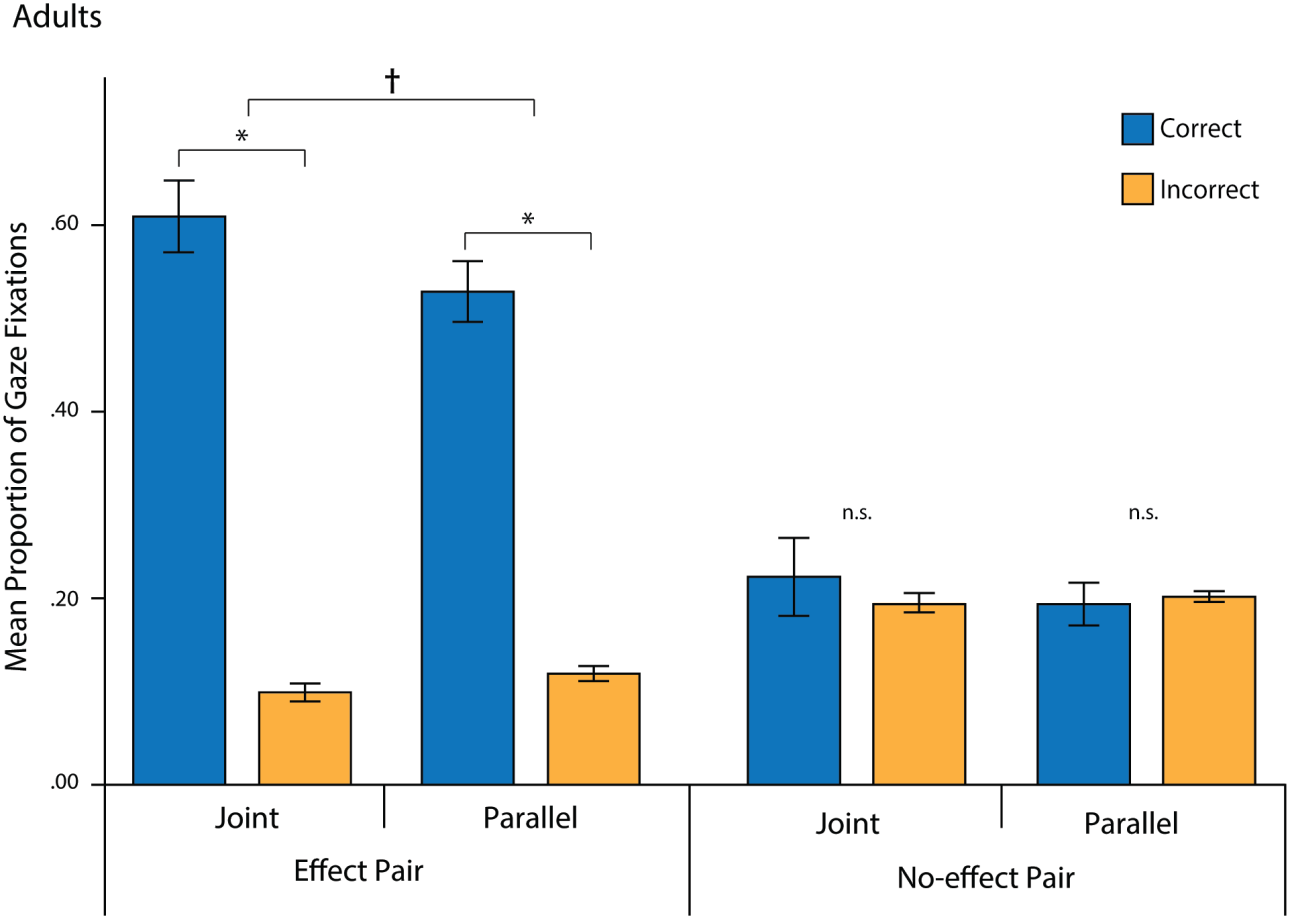
Adult eye-tracking results. Mean proportions of correct and incorrect fixations for each pair and condition. Error bars represent standard errors. *p < .001.

Correct and Incorrect proportions (Eqs 1 & 2) were entered into a repeated-measures analysis of variance (ANOVA) with Pair (Effect vs. No-effect) and Location (Correct vs. Incorrect) as within-subject factors and Condition (Joint vs. Parallel) as a between-subjects factor. This test yielded a significant interaction between Pair and Location, *F*(1,48) = 98.52, *p* < .001, *η*_*p*_^*2*^ = .67, and significant main effects of Location, *F*(1,48) = 141.19, *p* < .001, *η*_*p*_^*2*^ = .75, and Pair, *F*(1,48) = 98.50, *p* < .001, *η*_*p*_^*2*^ = .67 (Fig 2). Pairwise comparisons confirmed that adults made a higher proportion of correct than incorrect fixations for the Effect pair (*mean difference* = .46 [*SEM* = .03]; *p* < .001) but not for the No-effect pair (*mean difference* = .01 [*SEM* = .03]; *p* = .72). In addition, there was a marginal interaction between Location and Condition, *F*(1,48) = 3.01, *p* = .09, *η*_*p*_^*2*^ = .06. Based on our *a priori* hypotheses, planned comparisons were conducted to follow up on the interaction effect despite its marginal significance. These revealed marginally significant differences between conditions: descriptively, across pairs, adults in the Joint condition made more correct fixations (*mean difference* = .05, SEM = .03, *p* = .09) and fewer fixations to Incorrect locations (*mean difference* = -.01, SEM = .01, *p* = .09) than adults in the Parallel condition.

As fixations to the effect were only considered correct for the Effect pair, we controlled for differences in the number of correct regions between pairs by performing secondary analyses with looks to the effect excluded from all proportion scores. An ANOVA with Pair (Effect vs. No-effect) and Location (Correct vs. Incorrect) as within-subject factors and Condition (Joint vs. Parallel) as a between-subjects factor revealed a marginally significant effect of Location. Across pairs and conditions, proportions of correct fixations were descriptively greater than proportions of incorrect fixations, *F*(1,48) = 2.98, *p* = .09, *η*_*p*_^*2*^ = .06. There were no other main effects or interactions, *ps* > .16.

#### Learning performance

To investigate sensitivity to social context with respect to learning performance, participants were separated into high and low learning groups according to the median difference between correct and incorrect fixations, collapsed across pairs (including looks to the light for the Effect pair; *median* = .23, *SD* = .14). Based on this learning split, the mean difference score was .34 (*SD* = .11) for high learners and .12 (*SD* = .06) for low learners (*N* = 25 in each group).

**Fig 3:**
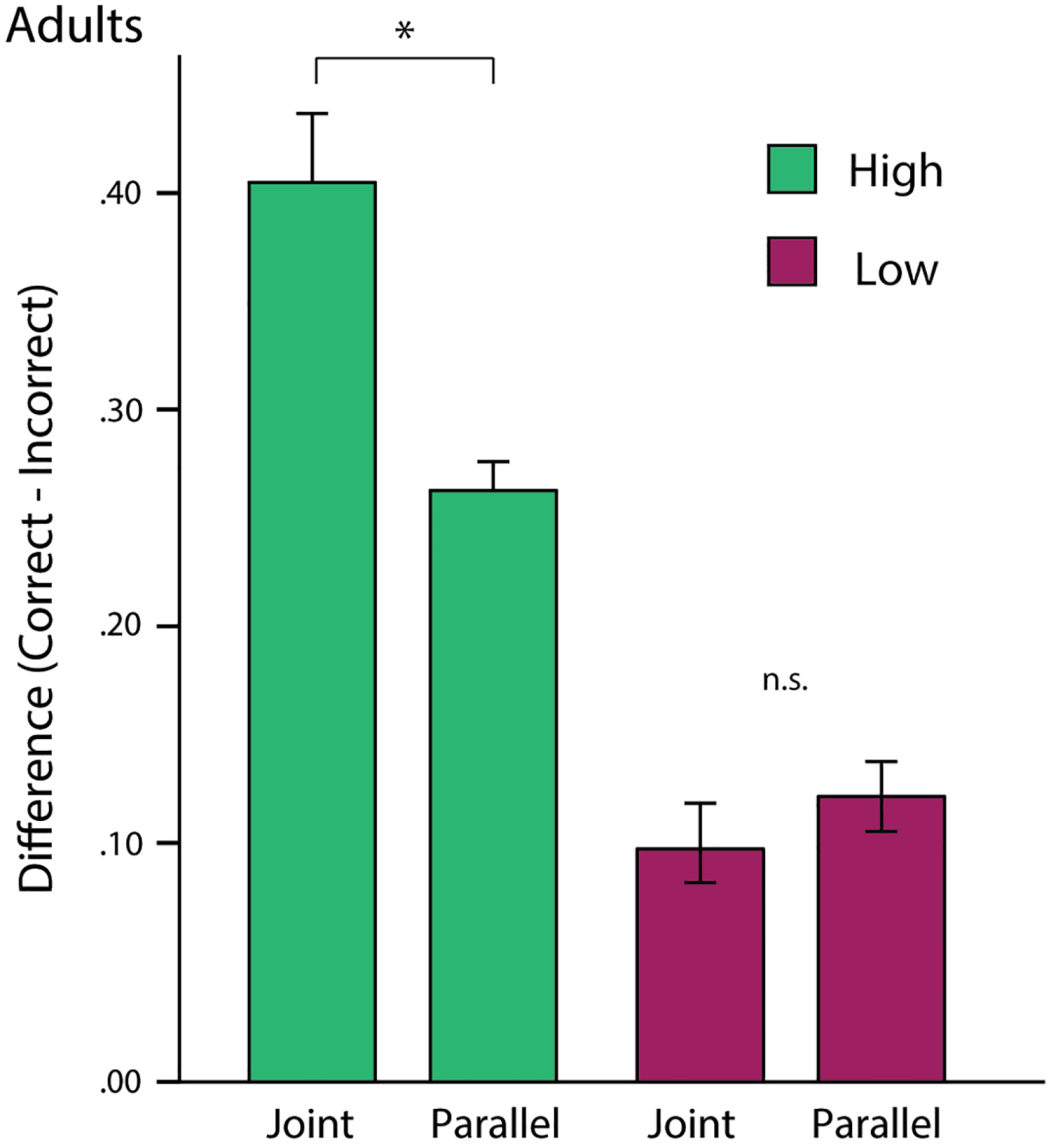
Adult eye-tracking results by learning group. Difference scores (Correct—Incorrect) by Learning groups (High vs. Low) and Condition, collapsed across pairs. Error bars represent standard errors.

Difference scores were entered into an ANOVA with Pair (Effect vs. No-effect) as a within-subjects factor, and Learning (High vs. Low) and Condition (Joint vs. Parallel) as between-subject factors. This yielded a significant interaction between Learning and Condition, *F*(1,46) = 16.08, *p* < .001, *η*_*p*_^*2*^ = .26 (Fig 3). Follow-up comparisons revealed that difference scores were significantly greater for the Joint relative to the Parallel condition for the high learners (*mean difference* = .14 [*SEM* = .03], *p* < .001), but not for low learners (*mean difference* = —.02 [*SEM* = .30], *p* = .42). There were no other interaction effects, *ps* > .27.

Consistent with the above analyses, there were also main effects of Pair, *F*(1,46) = 98.08, *p* < .001, *η*_*p*_^*2*^ = .68, and a main effect of Condition, *F*(1,46) = 8.09, *p* < .01, *η*_*p*_^*2*^ = .15. Difference scores were significantly higher for the Effect pair, relative to the No-effect pair, across groups (*mean difference* = .45 [*SEM* = .05], *p* < .001), indicating that adults made a higher proportion of correct relative to incorrect fixations for the Effect than the No-effect pair. The main effect of Condition reflected the fact that difference scores were significantly higher for the Joint relative to the Parallel condition when the learning groups were merged (*mean difference* = .06 [*SEM* = .02], *p* = .01); however, the interaction with Learning reveals that this main effect was driven by the large difference between conditions in the high learning group.

Low learners were not simply less attentive to the screen: an independent-samples t-test revealed no differences in overall gaze during the action observation task (*p* = .17) or during the social cue films (*p* = .96) between high and low learners. There were also no differences in visual attention to the action observation task (*p* = .54) or the social cue films (*p* = .88) between Joint and Parallel conditions. Thus, visual attention to the screen did not drive any of the observed patterns in predictive gaze.

### Action performance

#### Individual phase

The mean conditional probability, *P(B|A)*, across participants during the Individual phase was .28 (*SD* = .33) for the Effect pair and .18 (*SD* = .21) for the No-effect pair. An ANOVA with *P(B|A)* as the dependent variable, Pair (Effect and No-effect) as a within-subjects factor, and Condition (Joint vs. Parallel) as a between-subjects factor revealed a marginal main effect of Pair, *F*(1,34) = 3.49, *p* = .07, *η*_*p*_^*2*^ = .09 (Effect > No-effect). There were no other main effects or interactions, *ps* > .14.

We additionally tested whether action performance was different between high and low learners. *P(B|A)* was entered into an ANOVA with Pair (Effect vs. No-effect) as a within-subjects factor and Learning (High vs. Low) as a between-subjects factor. There were no main effects or interactions with Learning, *ps* > .43: high and low learners did not differ in their probability of performing action pairs.

#### Joint phase

Mean *P(B|A)* across participants during the Joint phase was .53 (*SD* = .42) for the Effect pair and .18 (*SD* = .21) for the No-effect pair. An ANOVA with *P(B|A)* as the dependent variable, Pair (Effect and No-effect) as a within-subjects factor, and Condition (Joint vs. parallel) as a between-subjects factor revealed no main effects or interactions, *ps* > .24.

Joint action performance also did not differ between high and low learners: an ANOVA with *P(B|A)* (Effect vs. No-effect) as a within-subjects factor and Learning (High vs. Low) as a between-subjects factor yielded no significant main effects or interactions with Learning, *ps* > .30.

#### Relations between anticipatory gaze and action performance

To identify relations between statistical learning during the action observation phase and spontaneous action performance, Spearman's rank-order correlations were performed between correct anticipations and the probability of performing an action pair in the Individual action context. These were conducted separately for each pair, across conditions. The correlation was not significant when including looks to the effect (*p* = .13) but there was a positive correlation between correct anticipations to the target action of the Effect pair—excluding looks to the effect—and the probability of performing an Effect pair, *r*_*s*_(43) = .29, *p* = .03. Participants who anticipated the target action correctly were thus more likely to perform the Effect pair themselves. There were no significant correlations for the No-effect pair, *ps* > .15. We did not assess correlations for the Joint action context because the first action was always performed by the experimenter rather than the participant.

### Toddlers

#### Eye-tracking results

**Fig 4:**
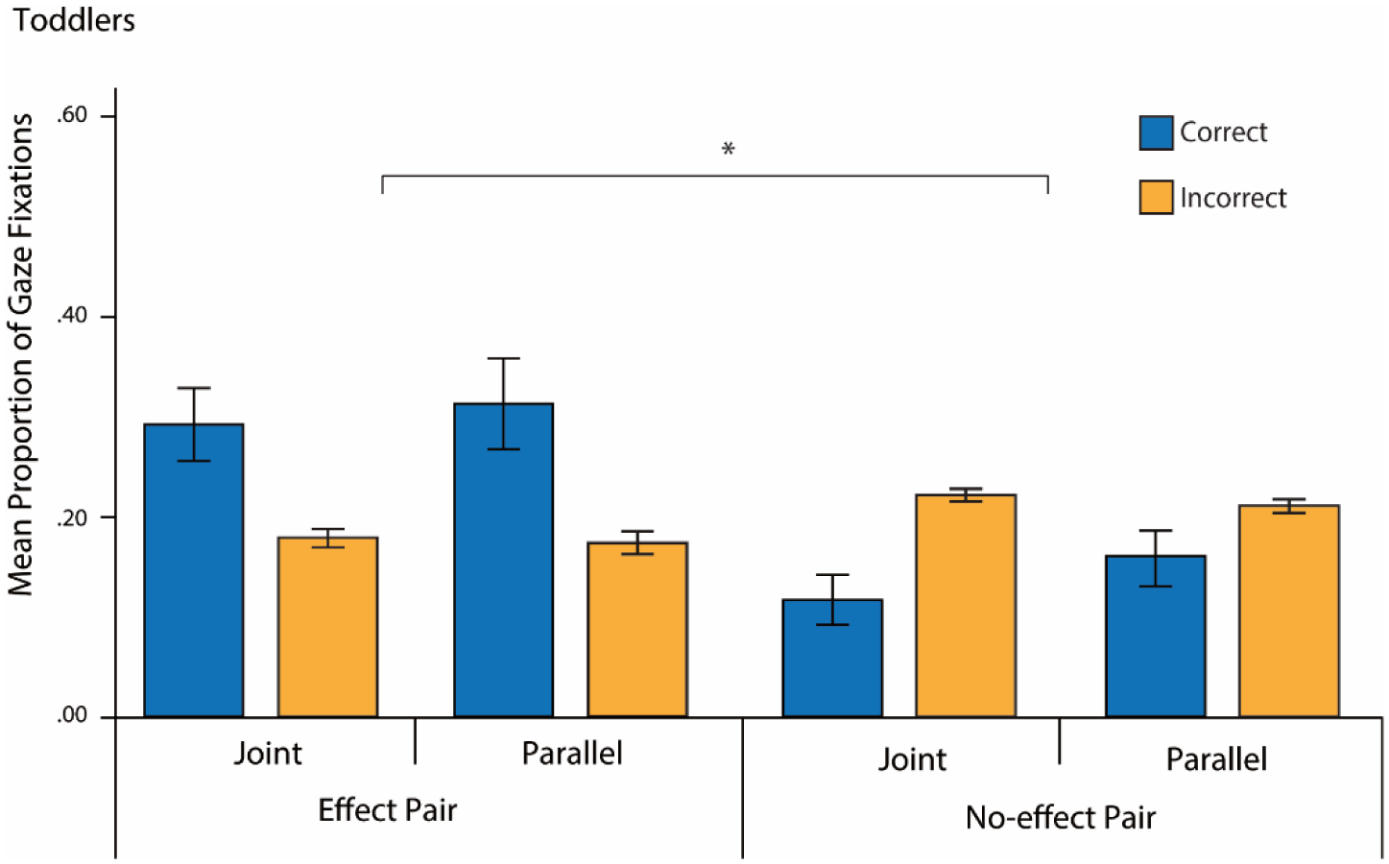
Toddler eye-tracking results. Mean proportions of correct and incorrect fixations for each pair and condition. The asterisk indicates an interaction between Pair and Location (Effect pair: C > I; No-effect pair: C < I across conditions). Error bars represent standard errors.

We conducted identical analyses for the toddler age group as for the adult group. Fig 4 illustrates the mean proportion of gaze fixations toddlers made during deterministic action pairs. An ANOVA with Pair (2: Effect vs. No-effect) and Location (2: Correct vs. Incorrect) as within-subject factors, and Condition (2: Joint vs. Parallel) as a between-subjects factor revealed a Pair by Location interaction, *F*(1,42) = 14.78, *p* < .001, *η*_*p*_^*2*^ = .26. Pairwise comparisons showed that toddlers made a higher proportion of correct than incorrect fixations for the Effect pair (*mean difference* = .08 [*SEM* = .03]; *p* = .002); in contrast, they made significantly fewer Correct than Incorrect fixations for the No-effect pair (*mean difference* = -.07 [*SEM* = .03]; *p* = .01). There were no main effects or interactions with Condition, *p*s > .31.

As before, we subsequently excluded looks to the effect as a correct location and only considered target object fixations. An ANOVA with Pair (Effect vs. No-effect) and Location (Correct vs. Incorrect) as within-subject factors, and Condition (2: Social vs. Non-social) as a between-subjects factor revealed a main effect of Location, *F*(1,42) = 19.20, *p* < .001, *η*_*p*_^*2*^ = .31 in the opposite direction: toddlers made fewer fixations to Correct relative to Incorrect locations across pairs (*mean difference* = -.06 [*SEM* = .02]; *p* < .001).

To assess the possibility that toddlers were simply looking to the location of the light effect throughout the action sequence, we conducted a secondary analysis on fixations to the effect only. The proportions of fixations to the light during the Effect pair were compared with proportions of fixations to the light during Random pairs (preceding the 2^nd^ action of the effect pair when it occurred elsewhere in the sequence). A paired-sample t-test revealed significant differences between the proportions (*mean difference* = .06, *SEM* = .03, *p* = .022) confirming that toddlers were not simply looking at the light throughout the action sequence. Rather, they seemed to selectively predict the light during the anticipatory time windows of the Effect pairs.

#### Learning performance

**Fig 5:**
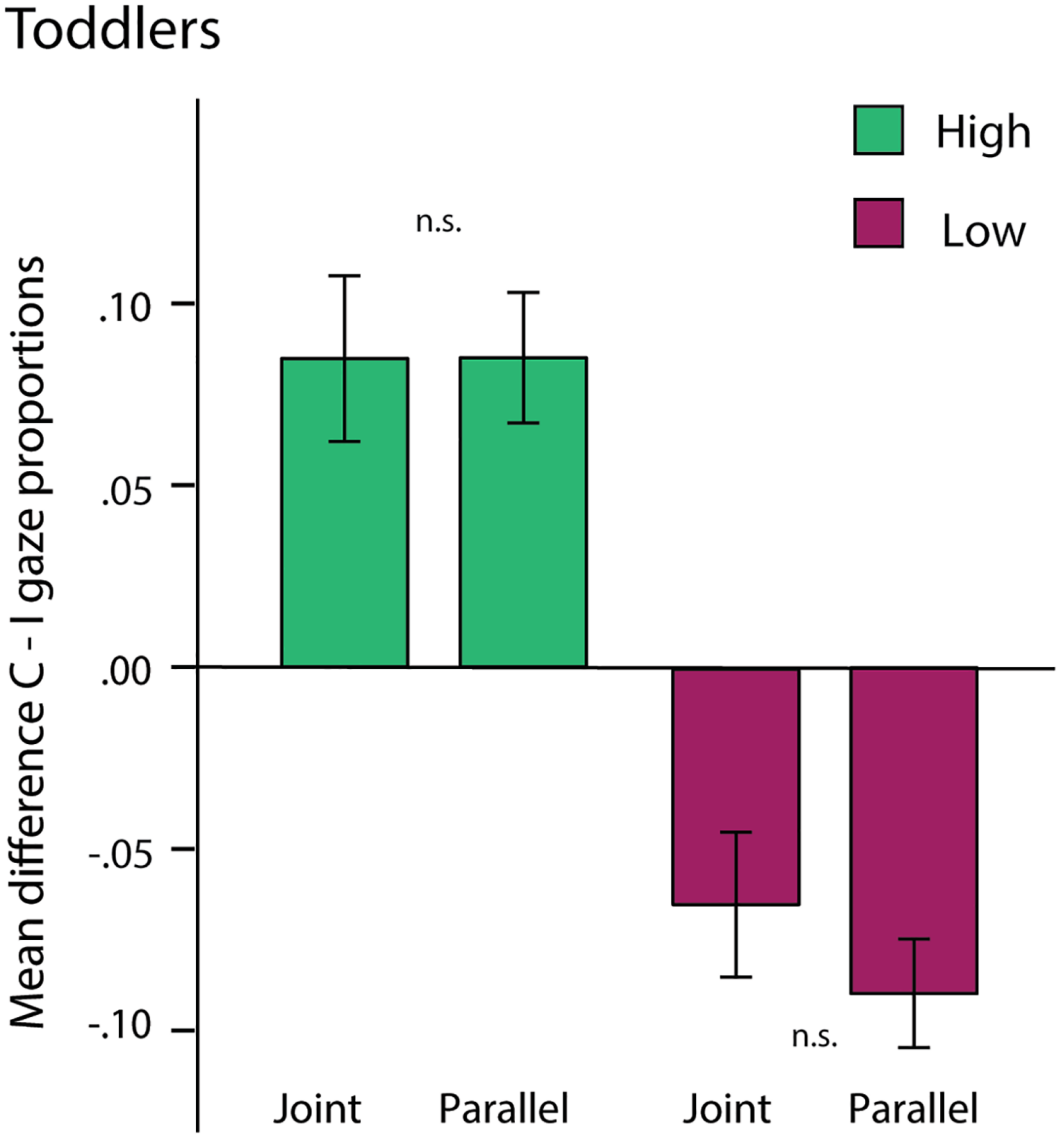
Toddler eye-tracking results by learning group. Difference scores (Correct—Incorrect) by Learning groups (High vs. Low) and Condition, across pairs for toddlers. Error bars represent standard errors.

Toddlers were also separated into high and low learners based on a median split of the mean difference score (correct–incorrect proportions) across pairs. The mean difference score was .09 (*SD* = .07) for high learners and -.08 (*SD* = .02) for low learners (*N* = 22 in each group). An ANOVA with Pair (Effect vs. No-effect) as a within-subjects factor and Learning (High vs. Low) and Condition (Joint vs. Parallel) as between-subjects factors showed no significant interaction effects with Learning, *ps* > .53 (Fig 5). Thus, toddlers did not differ in their visual anticipations according to whether they were in the Joint or Parallel conditions, regardless of learning performance.

Like the adults, toddlers also showed no differences in visual attention to the screen during the action observation task or the social cue task depending on whether they were high or low learners (*ps* = .55) There were also no differences in visual attention between Joint and Parallel conditions (*ps* > .47).

#### Action performance: Individual and Joint phases

Toddlers performed few actions during either action contexts. Table 2 contains the mean number of actions performed overall across Individual and Joint phases and the number of toddlers who performed an action pair. Across toddlers, there were no differences for *P(B|A)* based on Pair (Effect vs. No-effect) in either the Joint or the Individual action context, *ps* > .11. We conducted identical ANOVA analyses as we did for the adult group for consistency, though we did not expect toddlers’ performance data to yield meaningful results due to the low frequency of actions performed. There were no significant differences in between Joint or Parallel conditions for either pair in either the Individual or the Joint phases, *ps* > .48. There were no also no main effects or interactions with Learning, *ps* > .28, indicating that high and low learners did not differ in their probability of performing action pairs for either the Individual or Joint phases.

**Table 2:** Experimental session characteristics separated by age group.

Phase	Measure	Adults *(SD)*	Toddlers *(SD)*
**Action observation**	**Visual Attention**		
	Total looking time (*s*)	203.85 (*40*.*10*)	138.36 (*38*.*94*)
	Total fixations (no.)	70.28 (*20*.*17*)	54.61 (*19*.*20*)
	**Learning**		
	Difference *(Correct- Incorrect)* across pairs	0.23 (*0*.*14*)	0.01 (*0*.*10*)
**Action Performance**	**Individual phase:**		
	Mean # of actions performed	32.96 (*11*.*18*)	13.50 (*7*.*37*)
	P(B|A) Effect pair	.28 (.*33)*	.05 (.*14*)
	P(B|A) No-effect pair	.18 (.*21)*	.15 (.27)
	**Joint phase:**		
	P(B|A) Effect pair	0.53 (.*42)*	.09 (.*17)*
	P(B|A) No-effect pair	0.38 (.*37)*	.07 (.*14)*

The variables for each age group include measures from eye-tracking data (visual attention and learning) and post-observation play session (action performance).

#### Relations between anticipatory gaze and action performance

Correlations between the proportions of total correct fixations and the conditional probability *P(B|A)* of performing a pair were not significant for either Effect or No-effect pairs, *ps* > .14, whether looks to the light were included or not. Thus, there was no indication of any relation between toddlers’ predictive gaze fixations and their reproduction of action pairs in the individual action contexts.

## Discussion

Sensitivity to the regularities and structure contained within sequential, goal-directed actions is an important building block for generating expectations and understanding of the social actions we observe [7]. Until now, however, research on statistical learning for actions and research on joint action understanding have proceeded independently. The current study is the first to investigate the role of statistical learning abilities in tracking regularities across people, and whether these abilities are moderated by the social context defining the nature of the observed interaction.

We examined statistical learning skills of adults and toddlers during an action observation task. While keeping the observed sequence regularities identical, we manipulated the social context of the observed actions: two actors had either a joint action goal or their own individual goals. Consistent with previous research for individual actions, adults learned the statistical regularities across the two action partners: they made visual anticipations towards upcoming actions and reproduced the observed action pairs. However, learning only occurred when the regularity (i.e., the action pair) caused an effect; when there was no effect, adults did not anticipate the upcoming action within a pair even though it was statistically deterministic. Interestingly, we observed a difference in learning performance according to which social context the adults observed. That is, the adults correctly anticipated actions more—i.e., they learned better—if they observed the same actions in a joint rather than a parallel social context. For adults with lower performance, action context had no effect on learning.

Toddlers only learned the action pair with a corresponding action-effect and did not anticipate the target action of the pair that did not cause an effect. In addition, there was no evidence of any influence of the social manipulation on their visual predictions or self-produced actions, even for toddlers with better learning performance. In the following, we discuss these findings in more detail with respect to the different roles of social context, statistical learning, and action-effects for perception of social interactions in adults and young toddlers.

### Prediction in joint action: The role of action-effects for learning statistical regularities across action partners

Our first aim was to establish whether adults and toddlers could extract statistical regularities of actions across two action partners, given that they can do so for individual action sequences [33]. Our findings provide evidence that both age groups were able to correctly anticipate upcoming actions and their effects based on sequential regularities across the observed actors. Interestingly, both adults and toddlers only predicted the action pair that led to a salient effect. A relation between predictive gaze and self-produced actions was also only observed for this pair in the adult group.

These results are consistent with prior research on action-effect binding, which is situated within the framework of the ideomotor theory [25, 34, 35]. This theory proposes that perceiving an action-effect contingency creates bidirectional action-effect associations, and subsequent predictions are based on anticipation of the effect itself rather than the actor’s movements. In line with this framework, adults and toddlers in our experiment learned the structure of the action-effect pair (i.e. action *A*-action *B*-effect) and predicted the effect during observation of the first action in the pair.

Unexpectedly, adults and toddlers demonstrated essentially no learning for the No-effect pair. In contrast with evidence highlighting the automaticity and rapidity of visual statistical learning [36, 37], this finding suggests that exposure to the transitional probabilities between actions alone did not result in learning, as measured by anticipatory gaze. Traditional accounts of statistical learning propose that the underlying computations are based on transitional probabilities between items [5, 38]. Under this assumption, adults should be able to predict actions based on their statistical likelihood and not solely based on whether or not they cause an effect. In addition, a prior study showed that toddlers can predict upcoming target actions for individual action sequences [9]. One explanation for the lack of learning of the No-effect pair is that the saliency of the action-effect drew attention to the contingencies between the paired actions preceding it. Recent findings suggest that statistical learning, though robust, is constrained by features of the incoming information such as the surrounding perceptual environment and sensory modality [2, 30, 39]. Our stimuli presented complex visual information—i.e. two actors, effects, six different objects, a brief learning period—that could compete in terms of working memory or information processing. Adults and toddlers may have recruited a strategy that resulted in learning the more salient regularity within the sequence at the cost of not perceiving the second, less salient regularity. Toddlers, in fact, demonstrated more incorrect looks than correct looks for this pair, indicating that their performance was actually below chance. One explanation for their below-chance performance for the No-effect pair is that, in the absence of a visual effect, toddlers were free to engage in more visual exploratory behaviors to the other objects, potentially resulting in higher proportions of incorrect fixations for the No-effect pair relative to the Effect pair.

### The effect of social context on statistical learning

The second main aim of the current study was to investigate whether social context moderates how observers extract statistical regularities across two actors. Past research has demonstrated that subtle social cues, such as whether two actors share a joint intention or not, influence how subsequent actions are processed by onlookers [16, 21]. Eskenazi and colleagues [16] found activation in brain regions associated with organizing sequential movements towards a final goal, but only when participants observed two actors with a joint goal and not when they observed actors with parallel goals. Interestingly, activation in these regions only differed between Joint and Parallel conditions when participants were explicitly attending to the intention itself, and not during a working memory task. This finding suggests that processing joint goals depends on additional top-down input from brain regions involved in theory of mind and mental state reasoning.

In our study, we found that sensitivity to the social context only influenced prediction when adults were particularly skilled at picking up the statistical regularities. For those adults with better overall learning, we observed greater performance in the Joint relative to the Parallel condition; in contrast, there was no difference between conditions for adults who learned less well. One explanation for this effect is that statistical learning was only moderated by social context when the learning performance reached a certain threshold; put more simply, learning needed to be robust in the first place for the surrounding context to exert any influence. For the adults who were more proficient at learning statistical regularities, knowing that two people were acting together with a joint goal helped them to learn the regularities across their actions. If observing joint goals engages additional top-down neural processes [16, 40, 41], this might facilitate the detection of lower-level perceptual features of the action sequence such as statistical structure.

In a study on statistical learning of shape sequences, Abla and colleagues [31] similarly divided participants into learning groups based on performance in a post-test (i.e., familiarity judgements of the learned sequences). The better learners also showed earlier neural responses to the statistical regularities; less proficient learners only began to show neural responses to the regularities at the end of the learning phase. However, the less proficient learners were still above chance performance in the post-test, indicating that they still acquired retrospective sensitivity to the learned regularities even though they showed no neural markers of anticipation during observation. Thus, during learning, the ability to prediction future events may emerge more slowly than the ability to make retrospective judgments about what was observed. Our data also revealed variability in learning, as measured by online visual predictions, but perhaps both high and low learners would demonstrate similar performance in retrospective measures of learning which do not require predictions about the future (e.g., [39]). In a similar vein, sensitivity to social context was revealed in the online predictions of only the better learners, yet all observers—independent of learning proficiency—may still differentiate between the two social contexts in a retrospective measure such as a forced-choice or a familiarity test. This might also explain the lack of differences between conditions or learning groups observed in the action performance measures: despite varying degrees of success in predicting upcoming actions during the learning phase, these differences did not modulate their ability to reproduce the action pairs post-learning. The underlying sources of individual learning differences, and whether they relate more to general skills in detecting the visual regularities (as in Eskenazi et al. [16]) or in perceiving the social aspects of the task (as in Fawcett & Gredebäck [21]) is another question to address in future research.

An alternative interpretation that might explain the better performance in the Joint relative to the Parallel condition for the higher learners is that observing the parallel social cue actually hindered learning. Although there are situations in which two people who do not have a shared goal might still produce actions with statistical regularities (e.g., two people in competition with one another), it is perhaps less important for an observer to learn those regularities. Those who observed the two actors declare independent goals might have thus been biased away from detecting the regularities across their actions. A possible future study that could help answer this question would be to include a condition with no social context cue.

### Transferring learned regularities into action performance

The relation between predictive gaze behavior and action performance is an important indicator of whether observers are able to access knowledge they have acquired from observation and transfer it into their own action choices. This relation may be particularly important in a joint action context, as predicting a co-actor’s behavior and responding adaptively are necessary for smooth coordination [13]. In the adult group, predictive gaze was correlated with action performance: adults who made more predictive looks to the second actor’s actions also were more likely to reproduce the two-step action pair that caused an effect, even though they were acting alone. This suggests that observers were able to access knowledge of the sequence structure and use it for action control, but only when the actions led to a desired outcome or goal [9, 42]. For the toddler group, there were no correlations between predictive gaze and action performance. However, it is difficult to interpret the lack of a significant correlation as there were also far fewer data points during the action performance phase for the toddlers than for the adults.

### Statistical learning and social context in toddlerhood

Toddlers’ learning performance was not modulated by social context. These findings differ from those of Fawcett and Gredebäck [21], who showed that visual anticipations of 18-month-olds differ depending on the social relations established between two actors. In their study, toddlers were more likely to anticipate the joint goal of two actors in a Social condition than in a Non-social condition. However, in their experiment, the same action demonstration (moving a block to a final location in three steps) was performed in discrete, repetitive trials. Toddlers in the Social condition, relative to the Non-social condition, were more likely to bind the collaborative sequence together based on the social cue.

Despite the different outcomes, our results may complement these previous findings. In our design, statistically probable actions were embedded within a continuous stream of object-directed actions, with the regularities occurring across actors. Although we expected the social context to influence toddlers’ ability to learn the action structure, the action-effect instead determined whether or not they could successfully make action predictions. Perhaps the action-effect, rather than the social cue, determined whether toddlers perceived the interaction as having a shared joint goal. This notion is consistent with prior work showing that infants perceive sequences as collaborative when they result in an action outcome [21, 43]. In addition, the effect occurred repeatedly within the sequence itself, unlike the social cue, and may have been easier to retain in working memory.

Another point to consider is that, in the current study, we controlled for the spatial associations between actions and locations by rotating the stimuli between trial blocks so that the physical locations changed throughout the experiment. In the experiment by Fawcett and Gredebäck [21], location and goal were not dissociated; it is thus unknown whether infants were predicting an action goal or a spatial location. A recent study demonstrated that changing action locations, though more ecologically valid, makes action prediction more challenging for infants [44]. Here, given the changing target locations in every block, toddlers may have depended on the salient effect to successfully bind the two statistically coherent actions together. Faced with this more complex learning situation, toddlers may rely on easily identifiable information such as salient effects. Given a more extended learning opportunity or additional cues, toddlers might shift their learning strategy and use information from subtler cues such as eye gaze and dialogue. A promising avenue for future research would be to systematically disentangle the separate contributions of social cues and action-effects and identify how and when toddlers and young children can flexibly integrate these cues during action observation.

Finally, another possibility is that a greater degree of statistical learning is required for individual differences in sensitivity to social context to become apparent. That is, even the ‘high learners’ in the toddler group performed poorer in absolute terms than the ‘low learners’ in the adult group. As discussed above, subtle contextual cues may only influence learning above a certain threshold, which toddlers may not reach until later in development.

### Conclusions

Findings from the current study shed light on the relations between statistical learning, social context, and action-effects during observation of two action partners. Just as prior research has shown that people can detect regularities in observed actions, we present evidence that this ability extends to social situations in which regularities occur across two action partners. For adults who were good at extracting sequence regularities, subtle cues about the underlying intentions of two actors in a social context influenced their ability to detect these regularities. That is, when adults watched two actors sharing a joint action goal, they were more likely to detect regularities between their actions and predict an upcoming action than when they observed two actors with independent goals—even though the observed action streams were perceptually identical. Toddlers, at 18-months of age, showed similar learning performance regardless of the social context established between the two actors. These findings may reflect a developmental process, such that toddlers initially rely upon action-effects to define the social context between joint action partners, whereas social cues such as dialogue and gaze cues gain relevance later in development or under easier learning conditions.

## Supporting information

S1 File Dataset. This file contains the processed data used for statistical analyses.
(CSV)
